# Reconceptualizing Measurement of Emergency Contraceptive Use: Comparison of Approaches to Estimate the Use of Emergency Contraception

**DOI:** 10.1111/sifp.12111

**Published:** 2020-03-09

**Authors:** Elizabeth Larson, Antonia Morzenti, Georges Guiella, Peter Gichangi, Fredrick Makumbi, Yoonjoung Choi

## Abstract

Estimated use of emergency contraception (EC) remains low, and one reason is measurement challenges. The study aims to compare EC use estimates using five approaches. Data come from Performance Monitoring and Accountability 2020 surveys from 10 countries, representative sample surveys of women aged 15 to 49 years. We explore EC use employing the five definitions and calculate absolute differences between a reference definition (percentage of women currently using EC as the most effective method) and each of the subsequent four, including the most inclusive (percentage of women having used EC in the past year). Across the 17 geographies, estimated use varies greatly by definition and EC use employing the most inclusive definition is statistically significantly higher than the reference estimate. Impact of using various definitions is most pronounced among unmarried sexually active women. The conventional definition of EC use likely underestimates the magnitude of EC use, which has unique programmatic implications.

## BACKGROUND

There are an estimated 89 million unwanted pregnancies each year in low‐ and middle‐income countries, of which 211,000 end in maternal deaths (Guttmacher Institute 2017). Contraceptive use has been identified as one of the four pillars of the safe motherhood program, of which emergency contraception (EC) is a highly effective method (WHO 1996; Guttmacher Institute 2017). The most well‐known form of EC is the pill, although copper interuterine device insertion is also recognized. Contrary to other modern contraceptive methods, EC helps women prevent pregnancy after sexual intercourse in cases of forced sex, contraceptive failure, lack of use, or incorrect use (Westley et al. 2013). EC prevents or delays the egg from releasing from the ovary, however, it does not disrupt a pregnancy if the egg had already been fertilized (WHO and CCP 2018). The method is 95 percent effective if taken within 24 hours of sexual intercourse and prevents at least 50 percent of pregnancies within three days (Glasier et al. 2011; WHO 2018). EC represents a particularly appealing method for many subgroups of women, especially unmarried sexually active women. The UN Commission on Life‐Saving Commodities for Women and Children listed EC as one of its 13 “overlooked life‐saving commodities” that could save the lives of 6 million women and children (UNICEF 2012).

Despite EC's capacity for preventing unintended pregnancies and saving lives, it often remains inaccessible to women around the word, specifically those in low‐ and middle‐income countries. In many countries, ECs are available through private sector pharmacies, but due to limited consumer knowledge, potential users are not aware that post‐coital methods are available (UNICEF 2012). Further, there are often policy‐level barriers to access EC. Some countries require a prescription and/or pharmacies to have a special license to import EC, and opposition to ECs, often due to conflation with medical abortion, has made the product totally unavailable in others (Westley et al. 2013).

Unmarried sexually active women and women in their early twenties represent the two populations that exhibit the greatest use of EC (Morgan, Keesbury, and Speizer 2014a; Palermo, Bleck, and Westley 2014). The existing bias held by health workers against providing contraceptive methods to these two groups in many countries intensifies the difficulties in accessing EC (Bankole and Malarcher 2010; Sidze et al. 2014). Even in countries where there are no government‐ or provider‐imposed obstacles to procurement, stock outs are common (Dawson et al. 2015). The issue is further exacerbated by lack of guidance on how to improve commodity security and logistics for this important contraceptive method (Dawson et al. 2014).

Finally, the measurement and monitoring of EC use remains challenging. Contraceptive method use is typically measured using population‐based surveys, which typically leave interpretation of “current” open. Women may not report using EC currently since it is used neither during intercourse nor regularly. Previous research has identified similar challenges in accurately measuring coital‐dependent contraceptive use, such as rhythm, withdrawal, and condom use (Barden‐O'Fallon et al. 2014; Rossier, Senderowicz, and Soura 2014; Fabic and Becker 2017). Additionally, a conventional approach to understand and tabulate EC use is based on application of hierarchical method effectiveness during data analysis (ICF 2019). Thus, it can underestimate its prevalence when a woman uses EC in conjunction with more effective methods.

The measurement challenges around EC provide inadequate data, not capturing the level of EC users that are most programmatically relevant. Measuring the level of EC use differently for programmatic purposes is the essential first step to understanding correctly who uses EC, where, and their reasons for doing so. Also, inadequate measures can lead policies and programs to identify EC as a less utilized method than it actually is, resulting in fewer investments to ensure its accessibility. Such inadequate programming can create additional barriers disproportionately for subgroups that already experience difficulty in accessing contraceptives, such as unmarried sexually active women. Therefore, a lack of programmatically adequate data on EC use can result in resources not being directed toward where there is the greatest need.

To improve our understanding of EC use, this study aims to compare estimates of use based on five approaches, detailed below. Using nationally or subnationally representative population‐based survey data from 10 countries, we examine different estimates of EC use across the five approaches, among all women and among select subgroups of women. This approach allows us to estimate the number of additional EC users that are identified if survey questions change. Based on study findings, we recommend an additional survey question to measure EC use and programmatic implications for family planning as well as STI prevention.

## METHODS

### Data

Data come from the latest Performance Monitoring and Accountability 2020 (PMA2020) surveys. PMA2020 household and female surveys use a two‐stage cluster sample approach. Sampling clusters are selected using probability proportional to size within each strata, and a fixed number of households are selected randomly within each cluster. All women aged 15 to 49 years in sampled households are eligible and interviewed for female surveys, and, thus, the survey data are representative for a population. The female surveys collect data primarily on family planning and reproductive health, including contraceptive use, and have been conducted in 11 countries/geographies that have made commitment to achieve the FP2020 initiative. Most countries have national geographic coverage, but some countries conduct the surveys in only select geographies. Sample size varies by country, largely depending on the prevalence of modern contraceptive use (an indicator used for sample size calculation) and the number of strata in the country/geography.

Further information about PMA2020 survey methods and survey countries is available elsewhere (Zimmerman et al. 2017). This study includes the latest survey data from 7 countries in which data are nationally representative: Burkina Faso, Côte d'Ivoire, Ethiopia, Ghana, Kenya, Niger, and Uganda and from three countries in which data are representative at a subregional (e.g., state) level: Democratic Republic of Congo (DRC), Nigeria, and India (AAU SPH 2017; CRERD 2017; ICRH 2017; IIHMR 2017; INS Côte d'Ivoire 2017; INS Niger 2017; ISSP 2017; KNUST 2016; MUSPH 2018; Tulane SPHTM 2017). Table 1 shows the list of surveys, dates of data collection and samples sizes, by 17 geographic units used in this study.

**TABLE 1:** Latest PMA2020 surveys included in the study

Country/geography	Survey round^a^	Survey fieldwork (Year/month)	Number of households sampled	Number of female interviews completed
Burkina Faso	5	November to December 2017	2,906	3,556
Côte d'Ivoire	1	August to October 2017	2,548	2,785
Democratic Republic of Congo: Kinshasa	6	September to November 2017	1,914	2,590
Democratic Republic of Congo: Kongo Central	6	September to November 2017	1,716	1,703
Ethiopia	5	April to May 2017	7,730	7,464
Ghana	5	August to November 2016	4,182	3,746
India: Rajasthan	3	February to April 2017	5,136	6,095
Kenya	6	November to December 2017	6,342	5,913
Niger	4	May to September 2017	2,904	3,034
Nigeria: Anambra	4	March to April 2017	1,321	1,416
Nigeria: Kaduna	4	March to April 2017	2,278	2,860
Nigeria: Kano	4	March to April 2017	1,221	1,763
Nigeria: Lagos	4	March to April 2017	1,844	1,548
Nigeria: Nasarawa	4	March to April 2017	1,319	1,855
Nigeria: Rivers	4	March to April 2017	1,436	1,180
Nigeria: Taraba	4	March to April 2017	644	827
Uganda	6	April to May 2018	4,840	4,161

a A series of cross‐sectional surveys have been conducted, and it notes the survey round in each country.

PMA2020 collects information on women's awareness of contraceptive methods. Additionally, it collects data on current use and the most effective method used by a woman in the last 12 months (hereinafter referred to as recent use). For awareness of various contraceptive methods, respondents are probed with a description for each method (301g in Table 2). The probe for EC refers to the pill (“as an emergency measure after unprotected sexual intercourse women can take special pills at any time within three to five days to prevent pregnancy”), and, thus, EC in this study refers to the EC pill. Then, information regarding EC use is obtained through various questions as presented in Table 2 (Gates Institute 2018). In terms of measuring current EC use, most population‐based surveys use two questions (302a and 302b in Table 2). Among women who report that they or their partner are currently using a method, interviewers ask what the current method is and probe to determine if they use any other methods. Interviewers record all methods that respondents report (302b in Table 2).

**TABLE 2:** PMA2020 survey questions regarding emergency contraception

301g	Have you ever heard of emergency contraception? PROBE: As an emergency measure after unprotected sexual intercourse women can take special pills at any time within three to five days to prevent pregnancy
302a	[among women who are not pregnant] Are you or your partner currently doing something or using any method to delay or avoid getting pregnant?
302b	[among women who answered yes to 302a] Which method or methods are you using? Probe: Anything else? *Select all methods mentioned. Be sure to scroll to bottom to see all choices*.
306a	[among women who did not answer yes to 302a] In the last 12 months, have you ever done something or used a method to delay or avoid getting pregnant?
306b	[among women who answered yes to 3062a] Which method did you use most recently? Probe: Anything else? *Select most effective method (highest method on list). Scroll to bottom to see all choices*.
322a	[among women who answered EC in neither 302b nor 306b] Have you used emergency contraception at any time in the last 12 months? PROBE: As an emergency measure after unprotected sexual intercourse women can take special pills at any time within three to five days to prevent pregnancy.

NOTE: Full female “questionnaire” is available at: https://www.pma2020.org/sites/default/files/FQ-English-2017-11-15.pdf

The recent use questions (306a and 306b in Table 2), uniquely available in PMA2020, are designed to understand contraceptive dynamics among women who are not current users but have used in the past 12 months. The interviewer records the most recent method used, and in the event that two methods were used simultaneously, records only the most effective method. Therefore, if a woman reports that she recently used both injectables and EC, she is recorded as an injectable, but not EC, user.

Questions for both current and recent use, however, may underestimate EC use since women may forget to report the method because it is not used regularly or during sex. Furthermore, data that only collect information on the most effective contraceptive method will underestimate EC use if the method is used in conjunction with more effective methods. Additionally, the hierarchical recording of recent use responses even when respondents reported multiple methods (q306b in Table 2) limits potential analytical approaches that are available for current use responses, as described further below. Therefore, since late 2017, to overcome these limitations, an additional question has been introduced to ask about ever using EC in the last 12 month—with an explanation about EC to probe (322a in Table 2): “Have you used emergency contraception at any time in the last 12 months?”. PMA2020 asks this question to both pregnant and nonpregnant women. At the time of writing this paper, three surveys included in this report included the question: Burkina Faso 2017, Kenya 2017, and Uganda 2018.

### Measurement

We employed five definitions to measure EC use for this study, employing data from various questions regarding EC use described above. First is the commonly used conventional definition in most population‐based surveys, such as the Demographic and Health (DHS) Surveys: a woman is categorized as an EC user if EC is the most effective method that she reported using currently (Definition 1) (Croft, Marshal and Allen 2018). This definition is used in the context of assessing method mix, and questions 302a and 302b provide the data. Although information on multiple method use is available for further data analysis (302b), the most effective method is reported in method mix as a key survey result. Largely following the user effectiveness the methods hierarchy for analysis is: female sterilization, male sterilization, implants, IUD, injectable, pill, EC, male condom, female condoms, diaphragm, foam, beads, LAM, rhythm, withdrawal (Croft, Marshal and Allen 2018). In other words, if a woman reported using injectables and EC, she is counted as an injectable user, not an EC user in the method mix. This is the most restrictive, but also most widely used tabulation of EC use, and it is referred to as the conventional and reference definition in this report. Therefore, for the purpose of this paper, Definition 1 is used as a reference to understand the level of additional EC users identified by Definitions 2, 3, 4, and 5 in comparison to what is typically presented in the contraceptive method mix.

The second definition relaxes the method effectiveness hierarchy and disregards any other methods jointly used with EC: a woman is categorized as an EC user if she reported EC as a current method (Definition 2). Again, data for this measure come from responses to questions 302a and 302b. This definition does not affect the overall estimate of modern contraceptive prevalence (mCP) but can be considered a current method specific use rate in the population.

The third and fourth definitions includes women who recently used EC (i.e., in the past 12 months) as their most effective method—even if she is not currently using any method—to the first and second definitions, respectively (Table 3). Data for recent EC use come from questions 306a and 306b. In Definition 3, a woman is considered as an EC user if she reported EC as the most effective method that either she currently uses, or she used in the past 12 months. In Definition 4, a woman is classified as an EC user if she reported EC as a current method, regardless of other method use, or as the most effective method that she used in the past 12 months. Definition 3 is intended as a comparison to Definition 1 and Definition 4 as a comparison to Definition 2. Both definitions provide insight on the level of additional EC users that are identified when the definition of current use is expanded to the past 12 months.

**TABLE 3:** Women considered as emergency contraceptive users by definition

	Definition				
	1	2	3	4	5
Currently using emergency contraception (EC) as the most effective method	X	X	X	X	X
Currently using EC but not as the most effective method		X		X	X
Currently not using any methods but used EC as the most effective recent method in the past 12 months			X	X	X
Neither currently using EC nor having used EC as the most effective recent method in the past 12 months, but used EC in the past 12 months					X

The final definition, Definition 5, employs additional data from the new question, 322a. The question is asked to anyone who had not reported using EC in previous questions, 302b or 306b. A woman is categorized as an EC user: if she reported EC as a current method regardless of any other methods reported as well (from 302b); if she reported EC as the most effective method that she used in the past 12 months (from 306b); or if she reported ever using EC in the past 12 months (from 322a) Definition 5 is the most inclusive among the five measures, and intends to capture all women who ever used EC in the past 12 months. Table 3 summarizes differences across the definitions.

### Analysis

We conducted analyses for each of the 17 national or subnational geographies that have representative data for female population (Table 1). Using each definition, we calculate the percentage of women using EC, or the method specific use rate, and the 95 percent confidence interval, adjusted for sample design. The Wilson method was used to estimate the confidence interval, as the estimates are extremely low and it is the preferred method for asymmetrical confidence intervals for very high or low point estimates (Dean and Pagano 2015). Analyses are conducted among all *de facto* women aged 15 to 49 years.

Then, using Definition 1 as a reference, absolute differences in percentage point are calculated with each subsequent definition. Comparison between the reference and Definition 5 is available only from the three countries where a survey with the new question has been completed (Burkina Faso, Kenya, and Uganda), whereas comparison between the reference and the first three alternative definitions is available in all countries/geographies. Increases and decreases in EC use are considered significant if the 95 percent confidence intervals of results do not overlap (Gardner and Altman 1986).

Considering age and marital patterns of EC use, we conducted further analyses in four selected subgroups by marital status or age: women who are currently in union (i.e., married or living with a partner), women who are sexually active and currently not in union, women aged 15 to 19 years, women aged 15 to 24 years, and women aged 35 and older (Morgan, Keesbury, and Speizer 2014b).

## RESULTS

Across the 17 geographies, the majority of women are in union (range: 48.7 percent to 82.5 percent) or between the ages of 15 and 24 (range: 27.2 percent to 44.8 percent) (Appendix 1).1Appendixes are available at the supporting information tab at https://wileyonlinelibrary.com/journal/sfp. The percentage of unmarried sexually active women ranges from 0.7 percent to 24.1 percent.

### EC Use among All Women

**FIGURE 1:**
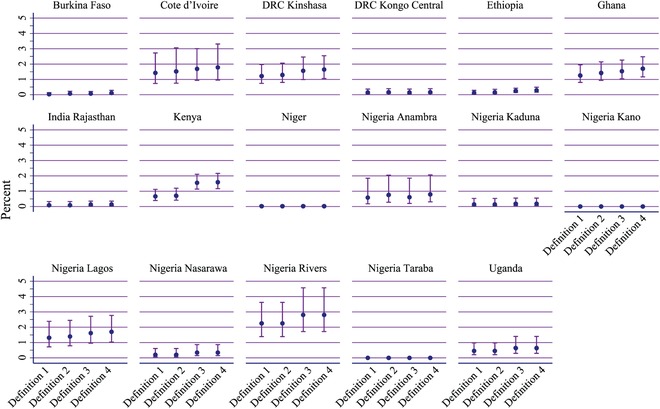
Percentage of all women using emergency contraception as estimated by Definitions 1, 2, 3, and 4 by geography NOTE: Vertical lines are the 95 percent confidence intervals and calculated using the Wilson method.

Using the conventional definition, the state of Rivers, Nigeria has the greatest percentage of EC users, with 2.2 percent of women (95 percent CI: 1.4, 3.6) (Figure 1 and Appendix 2). The two Nigerian states of Kano and Taraba have the lowest percentage, each having 0.0 percent of women using EC. Similar to Definition 1, the Rivers state of Nigeria has the greatest percentage of women using EC as measured by Definition 2 (D2: 2.2 percent, 95 percent CI: 1.4, 3.6). Additionally, Definition 2 picks up additional EC users in neither Kano nor Taraba, Nigeria, which both remain at 0.0 percent EC use.

Definitions 3 and 4 produce higher estimates of the percentage of women using EC (Figure 1), as they are more inclusive than Definitions 1 and 2. The geography with the greatest percentage of women using EC under Definition 3 is Rivers, Nigeria, with 2.8 percent (95 percent CI: 1.7, 4.6) of women using EC. Again, no women (0.0 percent) in Kano and Taraba, Nigeria use EC under Definition 3. Consistent with Definitions 1, 2 and 3, the state of Rivers, Nigeria has the greatest percentage of EC use with 2.8 percent (95 percent CI: 1.7, 4.6) and the states of Kano and Taraba have no women (0.0 percent) using EC.

**FIGURE 2:**
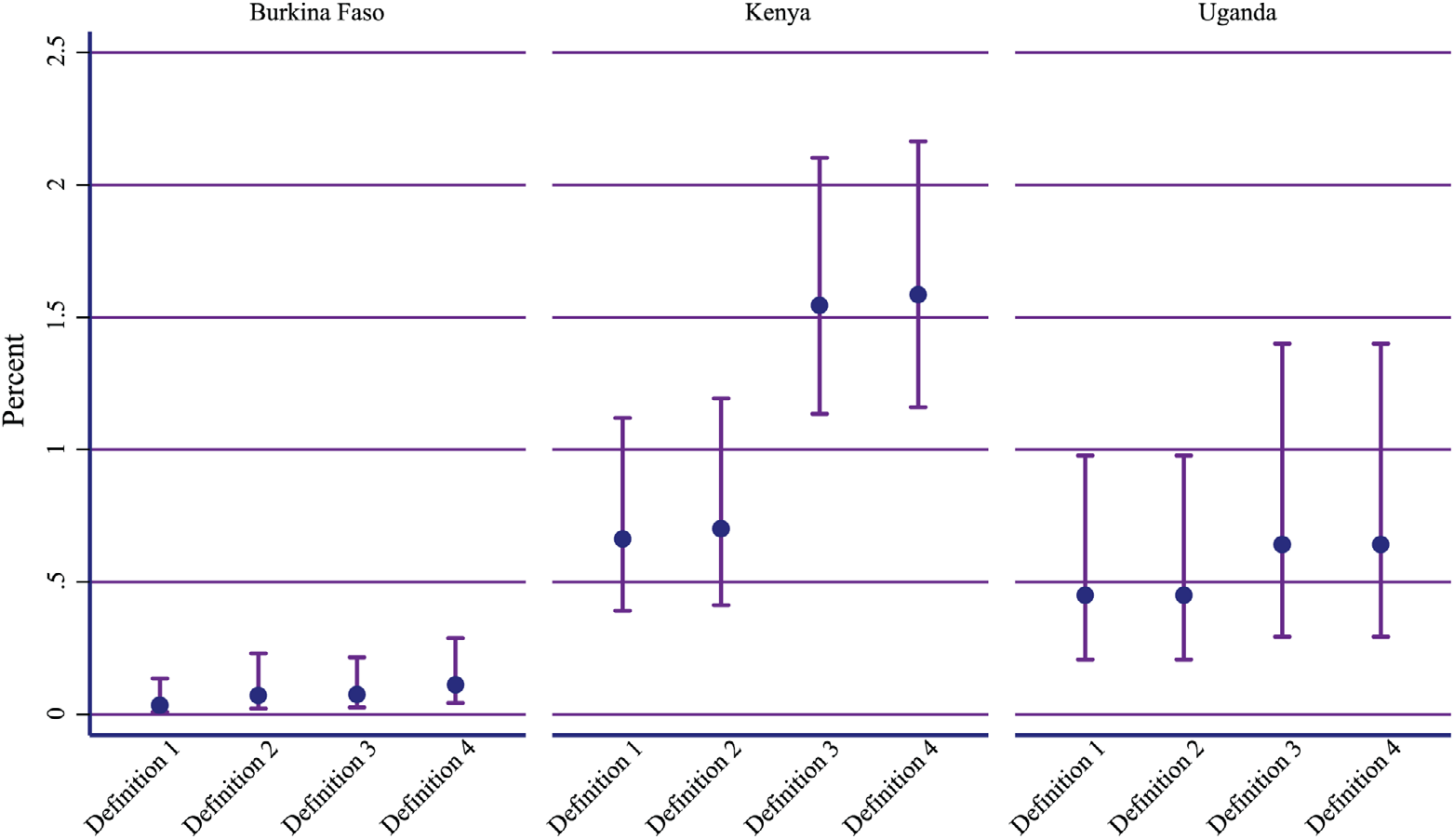
Comparison of the percentage of all women using emergency contraception as estimated by Definitions 1, 2, 3, and 4: Illustrative examples in Burkina Faso, Kenya, and Uganda NOTE: Vertical lines are the 95 percent confidence intervals calculated using the Wilson method.

Figure 2 focuses on the percentage estimates for Definitions 1, 2, 3, and 4 for Burkina Faso, Kenya, and Uganda, the three countries where data for Definition 5 exist. Among the three countries, Burkina Faso has the lowest percentage estimates for each of the definitions. Kenya has the highest percentage estimates for each of the definitions, and the percentage of EC users increases significantly when comparing Definition 4 to Definition 1 (D1: 0.7 percent, 95 percent CI: 0.4, 1.1; D4: 1.6, 95 percent CI: 1.2, 2.2). Finally, the percentage estimates for Uganda are between those for Burkina Faso and Kenya. Each geography shows a similar trend when comparing across definitions; Definition 1 and 2 are similar, with Definition 2 being higher—though not substantially, and Definition 3 and 4 are both higher than Definition 1 and 2, with Definition 4 being the greatest percentage estimate of use.

**FIGURE 3:**
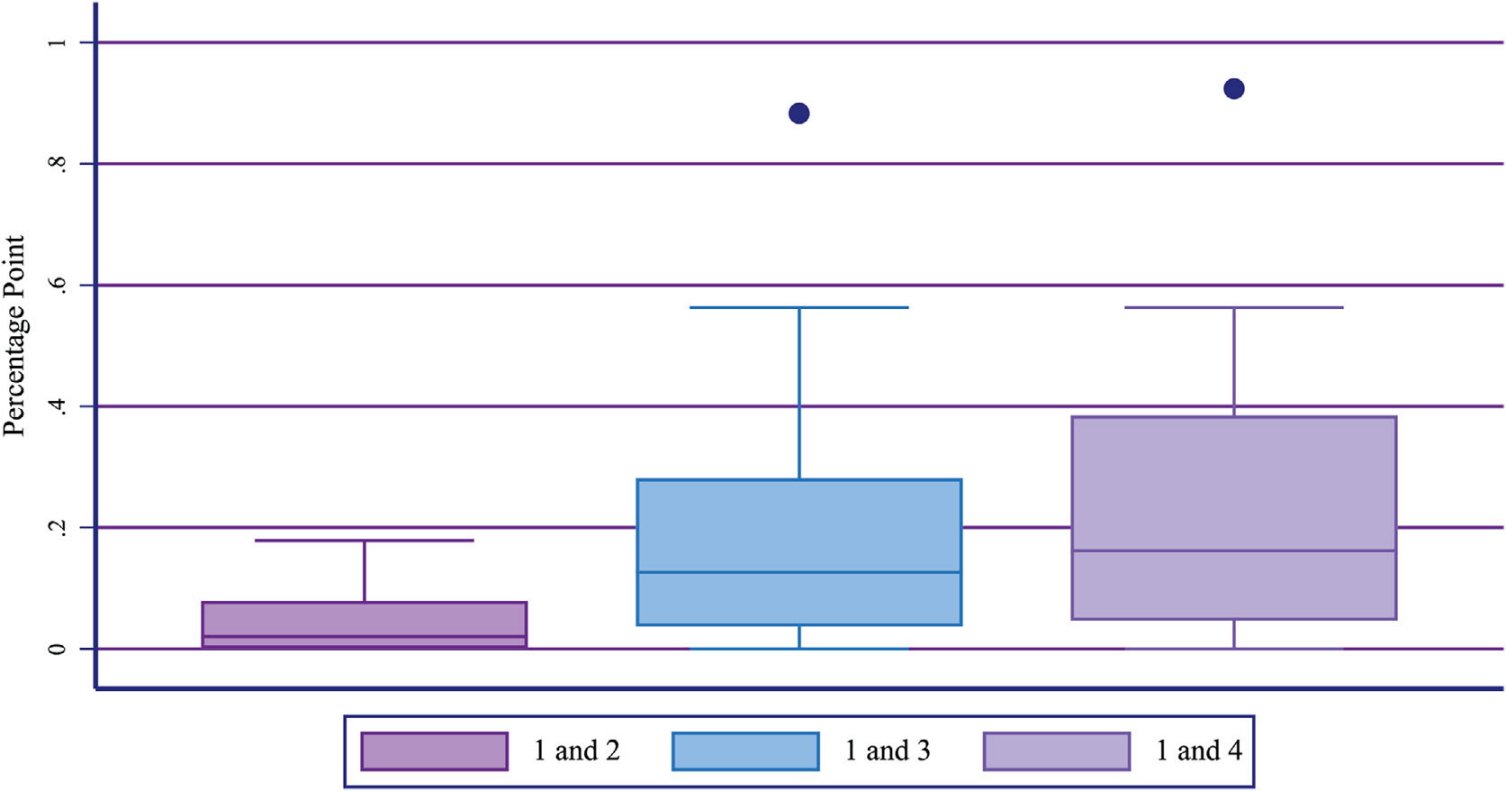
Distribution of percentage‐point difference in the percentage of all women using emergency contraception across 17 geographies: Based on different definitions between the reference (Definition 1) and Definitions 2, 3, and 4 NOTE: Box represents the interquartile range and the horizontal line in the center of the box is the median value.

Figure 3 depicts the distribution of percentage‐point increases between Definitions 1 and 2, Definitions 1 and 3, and Definitions 1 and 4 across all geographies. No comparison in any geography shows a difference exceeding 1 percentage point. The percentage‐point increase between Definitions 1 and 2 ranged from 0 in 12 geographies to 0.2 percentage points in Ghana and the Anambra State of Nigeria (Appendix 3). The percentage‐point increase between Definition 1 and 3 ranged from 0 in eight geographies to 0.9 in Kenya. Finally, the percentage‐point increase between Definitions 1 and 4 ranged from 0 in five geographies to 0.9 in Kenya.

### EC Use among Women in Subgroups

**FIGURE 4:**
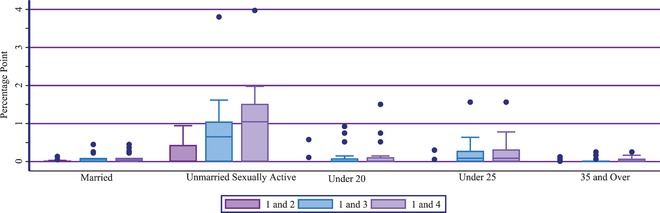
Distribution of percentage‐point difference in the percentage of married and unmarried sexually active women, women below 20 and below 25, and women 35 and above using emergency contraception across 17 geographies: Based on different definitions between the reference (Definition 1) and Definitions 2, 3, and 4 NOTE: Box represents the interquartile range and the horizontal line in the center of the box is the median value.

Figure 4 shows the distribution of percentage‐point increases between Definitions 1 and 2, Definitions 1 and 3 and Definitions 1 and 4 for each subgroup across all surveys. Among the five subgroups, unmarried sexually active women represent the group most sensitive to changes in measurement definitions. Among unmarried sexually active women, the percentage‐point increase between Definitions 1 and 2 ranged from 0 in 10 geographies to 0.9 in the Anambra State of Nigeria (Appendix 5). Additionally, the percentage‐point increase between Definitions 1 and 3 in EC use ranged from 0 in seven geographies to 3.8 in Kenya. Finally, the greatest percentage‐point increase between Definitions 1 and 4 for unmarried sexually active women was 4.0 in Kenya, a statistically significant change. Six geographies experienced no change between Definitions 1 and 4.

The married, below 20, below 25, and above 35 subgroups follow the same pattern as the unmarried sexually active subgroup, with the greatest percentage‐point increase being between Definitions 1 and 4, and the smallest increase being between Definitions 1 and 2 (Figure 4). For married women, the highest percentage‐point increase between Definitions 1 and 4 was 0.4 in both Ghana and the Rivers state of Nigeria (Appendix 5). The Nigerian state of Lagos had the greatest percentage‐point increase (1.5) between Definitions 1 and 4 for women below 20. Additionally, there was a statistically significant increase for this subgroup between Definition 1 and Definitions 3 and 4, where there was a 0.1 percentage‐point increase. The greatest percentage‐point increase between Definitions 1 and 4 in the below 25 subgroup of women was 1.6 in Kenya. Finally, the Kinshasa region of DRC had the greatest percentage‐point increase between Definitions 1 and 4 for women 35 and older (0.3).

### EC Use Incorporating 12‐Month Ever‐Use Data

Table 4 presents estimates of EC use among all women and by subgroup, using all five definitions, in Burkina Faso, Kenya, and Uganda. Across all three countries, Definition 1 provides the lowest percentage estimate of EC use, while Definition 5 provides the highest percentage estimate. Among the first four definitions for each of the three countries, as expected, removing method effectiveness hierarchy (Definitions 3 vs. 1, and 4 vs. 2) generally produces higher estimates than definitions with the hierarchy applied. However, the differences are generally small and not statistically significant. Including the recent most effective method to current methods (Definitions 2 vs. 1, and 4 vs. 3) resulted in minimal differences.

**TABLE 4:** Emergency contraception use based on Definitions 1 to 5, among all women and by subgroup: Burkina Faso, Kenya, and Uganda

	Definition 1		Definition 2	Definition 3	Definition 4	Definition 5	
Country	Estimate (%)	Confidence interval	Estimate (%)	Estimate (%)	Estimate (%)	Estimate (%)	Confidence interval
Burkina Faso							
All	0.0	0.0, 0.1	0.1	0.1	0.1	**1.2**	**0.8**, **1.7**
Married	0.0	0.0, 0.1	0.1	0.0	0.1	**0.9**	**0.6**, **1.5**
Unmarried sexually active	0.3	0.0, 1.5	0.3	0.5	0.5	**5.1**	**3.2**, **8.0**
Below 20	0.0	0.0, 0.3	0.0	0.0	0.0	**0.8**	**0.4**, **1.6**
Below 25	0.1	0.0, 0.3	0.1	0.1	0.1	**1.2**	**0.7**, **1.9**
35 and above	0.0	–	0.1	0.0	0.1	**0.7**	**0.4**, **1.4**
Kenya							
All	0.7	0.4, 1.1	0.7	1.5	1.6	**6.5**	**5.2**, **8.2**
Married	0.1	0.1, 0.3	0.2	0.4	0.4	**5.9**	**4.2**, **8.4**
Unmarried sexually active	2.6	1.4, 4.6	2.7	6.4	**6.5**	**13.6**	**10.8**, **16.9**
Below 20	0.8	0.4, 1.7	0.8	1.6	1.6	**4.3**	**3.2**, **5.9**
Below 25	1.1	0.6, 2.0	1.1	2.7	2.7	**6.1**	**4.9**, **7.6**
35 and above	0.1	0.0, 0.3	0.1	0.1	0.1	**4.6**	**3.0**, **6.9**
Uganda							
All	0.4	0.2, 1.0	0.4	0.6	0.6	**3.8**	**3.0**, **4.8**
Married	0.1	0.0, 0.3	0.1	0.1	0.1	**3.4**	**2.5**, **4.6**
Unmarried sexually active	2.4	1.1, 5.2	2.4	3.4	3.4	**8.8**	**6.4**, **12.2**
Below 20	0.4	0.1, 2.0	0.4	0.5	0.5	**1.7**	**0.9**, **3.4**
Below 25	0.7	0.3, 1.5	0.7	1.0	1.0	**3.5**	**2.6**, **4.6**
35 and above	0.1	0.0, 0.5	0.1	0.1	0.1	**3.2**	**2.1**, **4.9**

NOTE: Values in bold are statistically significant.

Among the three countries, Kenya has the highest percentage estimates of EC use for Definition 5 (Table 4). Note that 6.5 percent (95 percent CI: 5.2, 8.2) of all women report ever using EC in the past 12 months, which is a statistically significant increase over each of the other definitions. The percentage‐point increase between Definition 1 and Definition 5 in Burkina Faso is much smaller, however it is still a statistically significant increase over Definitions 1 through 4 (D1: 0.0 percent, 95 percent CI: 0.0, 0.1; D5: 1.2 percent, 95 percent CI: 0.8, 1.7). Finally, under Definition 1, 0.4 percent (95 percent CI: 0.2, 1.0) of all women in Uganda use EC. The percentage of users increases to 3.8 percent when using Definition 5 (95 percent CI: 3.0, 4.8). Similar to Burkina Faso and Kenya, Definition 5 is a statistically significant increase over the other definitions.

Among the five subgroups, unmarried sexually active women report the highest use of EC in all three countries, followed by women below 25. EC use across all definitions is lowest among women currently in union and women above the age of 35. In addition, the impact of using various definitions has similar patterns though with different magnitude across subgroups. In Kenya, for example, using Definition 1, 2.6 percent (95 percent CI: 1.4, 4.6) of unmarried sexually active women use EC (Table 4). The level, however, increases to 6.5 percent (95 percent CI: 4.7, 9.0) when using Definition 4, and 13.6 percent (95 percent CI: 10.8, 16.9) using Definition 5. Among women in union, although EC use is much lower than that among sexually active unmarried women, there is still a statistically significant difference when comparing Definition 5 (5.9 percent, 95 percent CI: 4.2, 8.4) to Definition 1 (0.1 percent, 95 percent CI: 0.1, 0.3), though with a smaller magnitude. For women below 20 and women below 25, 0.8 percent (95 percent CI: 0.4, 1.7) and 1.1 percent (95 percent CI: 0.6, 2.0) reported using EC under Definition 1, respectively. Similar to the pattern observed among married and unmarried sexually active women, there is a statistically significant change between Definitions 1 and 5, with 4.3 percent (95 percent CI: 3.2, 5.9) of women below 20, and 6.1 percent (95 percent CI: 4.9, 7.6) of women below 25 reporting EC use. Finally, the change between Definitions 1 and 5 for women above the age of 35 is also statistically significant, with 0.1 percent (95 percent CI: 0.0, 0.3) reporting EC use under Definition 1 and 4.6 percent (95 percent CI: 3.0, 6.9) under Definition 5. Burkina Faso and Uganda exhibited similar pattern across the subgroups.

## DISCUSSION

The purpose of this study was to compare the level of EC use using five different approaches. We find that the conventional approach to measure EC use, which only includes women who use EC as their most effective current method, does not adequately capture all EC users. In each country, as the definition of EC use broadens, the prevalence of EC user increases. The inclusion of all current EC users (Definition 2), and those who cite EC as their most effective recent method (Definitions 3 and 4) also misses a substantial percentage of women who use EC. Rather, the data support the conclusion that women often do not cite use of EC as a currently used contraceptive method when generally asked about methods they are using. Instead, as is demonstrated in Burkina Faso, Kenya, and Uganda, asking women directly about EC use in the last 12 months will result in a significant increase in the estimated prevalence EC use, and provides an estimate that potentially reflects the accurate EC use in a country.

Furthermore, the results support that the population with the greatest prevalence of EC use is unmarried sexually active women. When looking at EC use among this subgroup in all countries/geographies, the impact of using different definitions on the estimated level is magnified. In other words, asking unmarried sexually active women directly about her EC use in the past 12 months is much more effective at capturing EC use than the current conventional approach. The same is true in countries that have a higher estimate of EC use under the conventional definition, demonstrating that the effect of a targeted question on EC use is amplified in populations that have a high level of EC use in the conventional method mix.

The PMA2020 survey uses a primer question on awareness of all contraceptive methods before reporting use or nonuse of a method. The survey asks women whether they have heard of each specific method and provides a short probe to describe the method, however, the description does not include a list of brand names, which potentially leads to underreporting of the method. Still, the use of this series of questions reduces the likelihood that women are not reporting EC use because they do not remember that EC exists or do not consider EC to be a contraceptive method. Rather, the series of questions strengthens the conclusion that women do not often cite EC as a current contraceptive method.

Even though the results point toward an underestimation of the number of current EC users, they also support the notion that in many geographies, EC use remains extremely low (Dawson et al. 2015; Westley et al. 2013). In both the Kano and Taraba states of Nigeria, no EC users were identified, and EC use was estimated to be at 0.0 percent (greater than zero, but almost no users) in Niger under the most inclusive definition. Although this may indicate that EC use remains too low to measure in some geographies, Burkina Faso data suggest that asking more direct questions around EC use may be useful even in very low use settings, especially for certain subgroups. Similar to the three previously identified countries, almost no women reported using EC under the first four Definitions in Burkina Faso. However, when women were asked directly about their EC use, there was a significant increase in the proportion of users, indicating that EC may not actually be as low as is currently thought in many of these geographies.

This study is not without limitations. At this time, PMA2020 does not collect data on all recent contraceptive use, only the most effective current method. Therefore, this paper is unable to conclude whether the level of EC use would have been comparable between Definitions 4 and 5, if women are given the opportunity to report on all recent methods. Additionally, PMA2020 does not collect data on ever use of EC, and consequently, the paper is missing a potential sixth definition of EC use–ever use. However, the PMA2020 survey is currently the only large‐scale study that includes a separate question on EC in the past 12 months and therefore represents a key resource for future EC research. Due to the lack of additional surveys that include a separate question on EC in the past 12 months, a second limitation of the paper is its sole use of PMA2020 data. Therefore, future studies should aim to replicate this paper's results using a wider breadth of data once they become available. Finally, although PMA2020 does provide a primer question to help women to identify EC, the method's prevalence is based on self‐reporting and respondents may misreport. This problem is not unique to the PMA2020 survey and should be taken into account when drawing conclusions on self‐reported data.

The study's results demonstrate that it is advantageous to include a question on EC use in the last 12 months in surveys that are implemented in areas where there is a high prevalence of EC use. In areas of low EC use, the inclusion of a question may be too costly and will likely not pick up enough additional users to provide usable data for policy makers and program implementers. The DHS is including the 12 month EC question in the most recent version of their core questionnaire, which will provide more accurate estimates of EC use in their survey countries (DHS 2019). Additionally, given that there are similar challenges in the measurement of other short‐term methods, such as condoms, future research may want to focus on the potential benefits of the use of similar questions to identify additional users. Finally, identifying women who are pregnant at the time of the interview but also report using a short‐term method at any time in the last 12 months could provide additional insight into method failure or return to fertility after the discontinuation of a short‐term method.

These results also have important implications for both policy and programming. First, the findings show that EC is a significant resource, especially for unmarried sexually active women and adolescent girls, to prevent unplanned pregnancies that could otherwise lead to unsafe abortions or unwanted birth. To date, due to how EC is measured in surveys through the use of the conventional method, its use is underestimated. Therefore, the findings demonstrate the need for countries and programs to reevaluate their definition of EC use. Without accurate data on EC use, countries are unable to provide an adequate supply of EC to areas where there is a demand and decrease the number of unintended pregnancies, among unmarried sexually active women for example (Westley et al. 2013; Guttmacher Institute 2017). In addition, provision of EC can be an opportunity to advocate for dual method use for preventing sexually transmitted infections.

Finally, on a programmatic level, a more accurate definition will ensure that programs serve the populations most in need of EC, especially unmarried sexually active women. This is in line with current trends in family planning policy and programming, which in recent years have moved away from married women and toward those who are younger who practice sex outside of marriage (Williamson et al. 2009). The demand for EC, which is demonstrated by the results and was previously underestimated, shows the importance of increasing focus on EC delivery and the recognition of its use as an acceptable and highly effective contraceptive method.

## Supporting information

Supporting Information

## References

[sifp12111-bib-0001] [Dataset]Addis Ababa University School of Public Health, and The Bill and Melinda Gates Institute for Population and Reproductive Health at the Johns Hopkins Bloomberg School of Public Health . 2017. “Performance Monitoring and Accountability 2020 (PMA2020) Survey Round 5, PMA2017/Ethiopia‐R5.” Ethiopia and Baltimore, MD.

[sifp12111-bib-0002] Bankole, Akinrinola , and Shawn Malarcher . 2010 “Removing Barriers to Adolescents' Access to Contraceptive Information and Services.” Studies in Family Planning 41(2): 117–124. 21466111 10.1111/j.1728-4465.2010.00232.x

[sifp12111-bib-0003] Barden‐O'Fallon, Janine , Ilene S. Speizer , Lisa M. Calhoun , Livia Montana , and Priya Nanda . 2014 “Understanding Patterns of Temporary Method Use among Urban Women from Uttar Pradesh, India.” BMC Public Health 14(1): 1018. 25266733 10.1186/1471-2458-14-1018 PMC4190301

[sifp12111-bib-0004] [Dataset]Centre for Research Evaluation Resources and Development (CRERD) , Bayero University Kano (BUK), and The Bill and Melinda Gates Institute for Population and Reproductive Health at the Johns Hopkins Bloomberg School of Public Health . 2017. “Performance Monitoring and Accountability 2020 (PMA2020) Survey Round 4, PMA2017/Nigeria‐R4 (National).” Nigeria and Baltimore, MD.

[sifp12111-bib-0005] Croft, Trevor N. , Aileen M. J. Marshal , and Courney K. Allen . 2018. “Guide to DHS Statistics.” Rockville, MD.

[sifp12111-bib-0006] Dawson, A. , N. T. Tran , E. Westley E. , V. Mangiaterra , and M. Festin . 2014 “Improving Access to Emergency Contraception Pills through Strengthening Service Delivery and Demand Generation: A Systematic Review of Current Evidence in Low and Middle‐Income Countries.” PLoS ONE 9(10): e109315. 25285438 10.1371/journal.pone.0109315 PMC4186851

[sifp12111-bib-0007] Dawson, A. . 2015 “Workforce Interventions to Improve Access to Emergency Contraception Pills: A Systematic Review of Current Evidence in Low‐ and Middle‐Income Countries and Recommendations for Improving Performance.” BMC Health Services Research 15: 180. 25927734 10.1186/s12913-015-0815-2 PMC4421921

[sifp12111-bib-0008] Dean, Natalie , and Marcello Pagano . 2015 “Evaluating Confidence Interval Methods for Binomial Proportions in Clustered Surveys.” Journal of Survey Statistics and Methodology 3(4):484–503.

[sifp12111-bib-0009] Fabic, Madeleine Short , and Stan Becker . 2017 “Measuring Contraceptive Prevalence among Women Who are at Risk of Pregnancy.” Contraception 96(3): 183–188. 28666794 10.1016/j.contraception.2017.06.007

[sifp12111-bib-0010] Gardner, Martin J , and Douglas G. Altman . 1986 “Confidence Intervals Rather Than P Values: Estimation Rather Than Hypothesis Testing.” British Medical Journal (Clinical Research ed.)) 292(6522): 746–750. 3082422 10.1136/bmj.292.6522.746 PMC1339793

[sifp12111-bib-0011] Glasier, A. , S. T. Cameron , D. Blithe , B. Scherrer , H. Mathe , D. Levy , E. Gainer , and A. Ulmann . 2011 “Can We Identify Women at Risk of Pregnancy Despite using Emergency Contraception? Data from Randomized Trials of Ulipristal Acetate and Levonorgestrel.” Contraception 84(4): 363–367. 21920190 10.1016/j.contraception.2011.02.009

[sifp12111-bib-0012] Guttmacher Institute . 2017 Adding it Up: The Costs and Benefits of Investing in Sexual and Reproductive Health 2017. New York: Guttmacher Institute https://www.guttmacher.org/sites/default/files/factsheet/adding-it-up-contraception-mnh-2017.pdf. 15352318

[sifp12111-bib-0013] ICF . 2019 Demographic and Health Survey Interviewer's Manual. Rockville, MD: ICF.

[sifp12111-bib-0014] Indian Institute of Health Management Research (IIHMR) University in Jaipur, and The Bill and Melinda Gates Institute for Population and Reproductive Health at the Johns Hopkins Bloomberg School of Public Health . 2017. “Performance Monitoring and Accountability 2020 (PMA2020) Survey Round 3, PMA2017/India‐R3 (Rajasthan).” India and Baltimore, MD.

[sifp12111-bib-0015] [Dataset] Institut National de la Statistique de la Côte d'Ivoire, La Direction de Coordination du Programme National de Santé de la Mère et de l'Enfant, and The Bill and Melinda Gates Institute for Population and Reproductive Health at the Johns Hopkins Bloomberg School of Public Health . 2017. “Performance Monitoring and Accountability 2020 (PMA2020) Survey Round 1, PMA2017/Cote d'Ivoire‐R1. 2017.” Abidjan, Côte d'Ivoire and Baltimore, MD.

[sifp12111-bib-0016] [Dataset] Institut Supérieur des Sciences de la Population, Université Joseph Ki‐Zerbo, Ouagadougou, Burkina Faso, and The Bill and Melinda Gates Institute for Population and Reproductive Health at the Johns Hopkins Bloomberg School of Public Health . 2017. “Performance Monitoring and Accountability 2020 (PMA2020) Survey Round 5, PMA2017/Burkina Faso‐R5.” Ouagadougou, Burkina Faso and Baltimore, MD.

[sifp12111-bib-0017] [Dataset] International Centre for Reproductive Health Kenya, and The Bill and Melinda Gates Institute for Population and Reproductive Health at the Johns Hopkins Bloomberg School of Public Health . 2017. “Performance Monitoring and Accountability 2020 (PMA2020) Survey Round 6, PMA2017/Kenya‐R6.” Kenya and Baltimore, MD.

[sifp12111-bib-0018] Kwame Nkrumah University of Science & Technology School of Medicine, and The Bill and Melinda Gates Institute for Population and Reproductive Health at the Johns Hopkins Bloomberg School of Public Health . 2016. “Performance Monitoring and Accountability 2020 (PMA2020) Survey Round 5, PMA2016/Ghana‐R5. 2016.” Ghana and Baltimore, MD.

[sifp12111-bib-0019] [Dataset] Makerere University School of Public Health at the College of Health Sciences, and The Bill and Melinda Gates Institute for Population and Reproductive Health at the Johns Hopkins Bloomberg School of Public Health . 2018. “Performance Monitoring and Accountability 2020 (PMA2020) Survey Round 6, PMA2018/Uganda‐R6.” Uganda and Baltimore, MD.

[sifp12111-bib-0020] Morgan, Gwendolyn , Jill Keesbury , and Ilene Speizer . 2014a “Characteristics and Patterns of Use of Emergency Contraception among Urban Women in Nigeria and Kenya.” Studies in Family Planning 45(1): 59–72. 24615575 10.1111/j.1728-4465.2014.00376.x PMC4230484

[sifp12111-bib-0021] Morgan, Gwendolyn . 2014b “Emergency Contraceptive Knowledge and Use among Urban Women in Nigeria and Kenya.” Studies in Family Planning 45(1): 59–72. 24615575 10.1111/j.1728-4465.2014.00376.x PMC4230484

[sifp12111-bib-0022] [Dataset] Niger/Niamey Institut National de la Statistique (National Institute of Statistics), and The Bill and Melinda Gates Institute for Population and Reproductive Health at the Johns Hopkins Bloomberg School of Public Health . 2017. “Performance Monitoring and Accountability 2020 (PMA2020) Survey Round 4, PMA2017/Niger‐R4 (National).” Niamey, Niger and Baltimore, MD.

[sifp12111-bib-0023] Palermo, Tia , Jennifer Bleck , and Elizabeth Westley . 2014 “Knowledge and Use of Emergency Contraception: A Multicountry Analysis.” International Perspectives on Sexual and Reproductive Health 40(2): 79–86. 25051579 10.1363/4007914

[sifp12111-bib-0024] Rossier, Clémentine , Leigh Senderowicz , and Abdramane Soura . 2014 “Do natural Methods Count? Underreporting of Natural Contraception in Urban Burkina Faso.” Studies in Family Planning 45(2):171–182. 24931074 10.1111/j.1728-4465.2014.00383.x

[sifp12111-bib-0025] Sidze, Estelle M , Solène Lardoux , Ilene S Speizer , Cheikh M Faye , Michael M Mutua , and Fanding Badji . 2014 “Young Women's Access to and Use of Contraceptives: The Role of Providers' Restrictions in Urban Senegal.” International Perspectives on Sexual and Reproductive Health 40(4): 176–183. 25565345 10.1363/4017614 PMC6652199

[sifp12111-bib-0026] The Bill and Melinda Gates Institute for Population and Reproductive Health at the Johns Hopkins Bloomberg School of Public Health . 2018 Performance Monitoring and Accountability 2020 (PMA2020) Female Questionnaire. Baltimore, MD.

[sifp12111-bib-0027] The DHS Program . 2019. “DHS Model Questionnaire ‐ Phase 8.” Rockville, MD.

[sifp12111-bib-0028] [Dataset] Tulane University School of Public Health, University of Kinshasa School of Public Health, and The Bill and Melinda Gates Institute for Population and Reproductive Health at the Johns Hopkins Bloomberg School of Public Health . 2017. “Performance Monitoring and Accountability 2020 (PMA2020) Survey Round 6, PMA2017/DRC‐R6 (Kinshasa & Kongo Central).” Kinshasa, DRC and Baltimore, MD.

[sifp12111-bib-0029] United Nations International Children's Emergency Fund . 2012. “UN Commission on Life‐Saving Commodities for Women and Children.” New York, NY.

[sifp12111-bib-0030] Westley, E. , N. Kapp , T. Palermo , and J. Bleck . 2013 “A Review of Global Access to Emergency Contraception.” International Journal of Gynecology & Obstetrics 123(1): 4–6. 23856676 10.1016/j.ijgo.2013.04.019

[sifp12111-bib-0031] Williamson, L. M. , A. Parkes , D. Wight , M. Petticrew , and G. J. Hart . 2009 “Limits to Modern Contraceptive Use among Young Women in Developing Countries: A Systematic Review of Qualitative Research.” Reproductive Health 6(3): 3. 19228420 10.1186/1742-4755-6-3 PMC2652437

[sifp12111-bib-0032] World Health Organization (WHO) . 1996. “Mother‐Baby Package: Implementing Safe Motherhood in Countries: Practical Guide.”

[sifp12111-bib-0033] World Health Organization (WHO) . 2018. “Emergency Contraception.”

[sifp12111-bib-0034] World Health Organizaiton (WHO ) Department of Reproductive Health and Research, Johns Hopkins Bloomberg, and School of Public Health/Center for Communication Programs (CCP) . 2018. “Family Planning: A Global Handbook for Providers (2018 Update).” Baltimore, MD; Geneva, Switzerland.

[sifp12111-bib-0035] Zimmerman, Linnea , Hannah Olson , PMA Principal Investigators Group , Amy Tsui , and Scott Radloff . 2017 “PMA2020: Rapid Turn‐Around Survey Data to Monitor Family Planning Service and Practice in Ten Countries.” Studies in Family Planning 48(3): 293–303. 28885679 10.1111/sifp.12031 PMC6084342

