# Supplementary material for: Reconceptualizing Measurement of Emergency Contraceptive Use: Comparison of Approaches to Estimate the Use of Emergency Contraception
Source: Stud Fam Plann. 2020 Mar 9;51(1):87–102. doi: 10.1111/sifp.12111 (PMC7187174; doi:10.1111/sifp.12111)
Supplement: Supplementary file 1 — Supporting Information [file SIFP-51-87-s001.docx]

T1 Background Characteristics of female sample by geography

|  | | **Burkina Faso** | | | **Côte d’Ivoire** | | | **Democratic Republic of Congo** | | | | | | **Ethiopia** | | **Ghana** | | | **India Rajasthan** | | | **Kenya** | | **Niger** | | | **Nigeria** | | | | | | | | | | | | | | | | **Uganda** | |
| --- | --- | --- | --- | --- | --- | --- | --- | --- | --- | --- | --- | --- | --- | --- | --- | --- | --- | --- | --- | --- | --- | --- | --- | --- | --- | --- | --- | --- | --- | --- | --- | --- | --- | --- | --- | --- | --- | --- | --- | --- | --- | --- | --- | --- |
|  |  |  |  |  |  |  |  | **Kinshasa** | | | **Kongo Central** | | |  |  |  |  |  |  |  |  |  |  |  |  |  | **Anambra** | | | **Kaduna** | | **Kano** | | **Lagos** | | **Nasarawa** | | **Rivers** | | | **Taraba** | |  |  |
|  | | *n* | | *%* | *n* | | *%* | *n* | *%* | | *n* | *%* | | *n* | *%* | *n* | *%* | | *n* | *%* | | *n* | *%* | *n* | | *%* | *n* | | *%* | *n* | *%* | *n* | *%* | *n* | *%* | *n* | *%* | *n* | | *%* | *n* | *%* | *n* | *%* |
| All Women | | 3556 | | – | 2785 | | – | 2590 | – | | 1703 | – | | 7464 | – | 3746 | – | | 6095 | – | | 5913 | – | 3034 | | – | 1416 | | – | 2860 | – | 1763 | – | 1548 | – | 1855 | – | 1180 | | – | 827 | – | 4161 | – |
| Marital Status | | | | | | | | | | | | | | | | | | | | | | | | | | | | | | | | | | | | | | | | | | | | |
| Married | | 2629 | | 74.9 | 1760 | | 64.3 | 1252 | 48.8 | | 1143 | 67.7 | | 4857 | 66.0 | 2132 | 57.9 | | 4551 | 75.4 | | 3400 | 57.9 | 2491 | | 82.5 | 683 | | 48.7 | 2242 | 78.5 | 1304 | 74.1 | 1009 | 65.7 | 1201 | 65.0 | 691 | | 59.4 | 557 | 68.1 | 2697 | 63.8 |
| Unmarried Sexually Active | | 303 | | 8.6 | 558 | | 20.4 | 619 | 24.1 | | 295 | 17.5 | | 373 | 5.1 | 702 | 19.1 | | 43 | 0.7 | | 1091 | 18.6 | 71 | | 2.4 | 265 | | 18.9 | 117 | 4.1 | 38 | 2.1 | 167 | 10.9 | 253 | 13.7 | 214 | | 18.4 | 130 | 15.9 | 700 | 16.6 |
| Age | | | | | | | | | | | | | | | | | | | | | | | | | | | | | | | | | | | | | | | | | | | | |
| 15-19 | | 792 | | 22.4 | 574 | | 21.4 | 566 | 24.2 | | 373 | 21.6 | | 1768 | 23.7 | 708 | 18.9 | | 1173 | 19.0 | | 1255 | 21.6 | 679 | | 21.5 | 303 | | 22.3 | 700 | 23.1 | 419 | 23.5 | 218 | 14.1 | 422 | 20.7 | 194 | | 16.6 | 171 | 21.0 | 919 | 21.7 |
| 20-24 | | 638 | | 17.0 | 529 | | 19.8 | 513 | 20.6 | | 262 | 15.4 | | 1333 | 16.5 | 678 | 18.5 | | 1164 | 19.8 | | 1117 | 19.0 | 553 | | 19.7 | 221 | | 16.1 | 551 | 20.2 | 325 | 18.8 | 203 | 13.1 | 341 | 17.9 | 209 | | 17.5 | 149 | 18.4 | 894 | 21.5 |
| 25-29 | | 612 | | 17.0 | 511 | | 19.2 | 455 | 16.7 | | 263 | 15.2 | | 1410 | 18.9 | 627 | 17.2 | | 1003 | 16.4 | | 1057 | 18.1 | 523 | | 18.2 | 220 | | 14.5 | 509 | 18.1 | 283 | 16.2 | 257 | 16.7 | 355 | 20.2 | 192 | | 18.3 | 175 | 21.3 | 747 | 17.7 |
| 30-34 | | 505 | | 14.4 | 416 | | 15.1 | 351 | 12.3 | | 264 | 16.0 | | 1040 | 14.7 | 567 | 15.5 | | 910 | 14.7 | | 891 | 14.9 | 413 | | 13.1 | 223 | | 15.8 | 431 | 15.6 | 262 | 14.7 | 306 | 20.3 | 289 | 16.6 | 209 | | 19.0 | 116 | 13.6 | 578 | 13.8 |
| 35-39 | | 393 | | 11.7 | 312 | | 10.8 | 274 | 10.7 | | 223 | 12.4 | | 880 | 12.8 | 459 | 12.2 | | 725 | 12.2 | | 639 | 10.5 | 385 | | 12.2 | 166 | | 12.9 | 272 | 9.1 | 195 | 11.1 | 280 | 17.6 | 200 | 11.6 | 190 | | 14.8 | 98 | 12.1 | 485 | 11.8 |
| 40-44 | | 340 | | 10.6 | 230 | | 8.2 | 245 | 9.2 | | 162 | 10.0 | | 541 | 7.9 | 364 | 10.0 | | 576 | 9.9 | | 540 | 9.4 | 297 | | 10.2 | 147 | | 10.2 | 208 | 7.8 | 158 | 9.1 | 162 | 10.8 | 122 | 6.6 | 106 | | 8.5 | 62 | 7.4 | 321 | 7.4 |
| 45-49 | 232 | | 6.9 | | 166 | 5.5 | | 164 | | 6.4 | 142 | | 9.3 | 389 | 5.5 | 280 | | 7.6 | 483 | | 8.0 | 377 | 6.4 | 170 | 5.0 | | 123 | 8.3 | | 184 | 6.2 | 118 | 6.7 | 109 | 7.3 | 117 | 6.3 | 63 | 5.3 | | 47 | 6.3 | 281 | 6.1 |

T2 Percent estimate of emergency contraception use among all women by definition and geography, bolded values are statistically significant

|  |  | **Definition 1** | | **Definition 2** | | **Definition 3** | | **Definition 4** | |
| --- | --- | --- | --- | --- | --- | --- | --- | --- | --- |
| **Country/ Geography** | **N** | **Percent** | **CI** | **Percent** | **CI** | **Percent** | **CI** | **Percent** | **CI** |
| **Burkina Faso** | 3512 | 0.0 | 0.0, 0.1 | 0.1 | 0.0, 0.2 | 0.1 | 0.0, 0.2 | 0.1 | 0.0, 0.3 |
| **Côte d’Ivoire** | 2738 | 1.4 | 0.7, 2.7 | 1.5 | 0.8, 3.1 | 1.7 | 0.9, 3.0 | 1.8 | 1.0, 3.3 |
| **DRC: Kinshasa** | 2568 | 1.2 | 0.7, 2.0 | 1.3 | 0.8, 2.1 | 1.6 | 1.0, 2.5 | 1.6 | 1.1, 2.5 |
| **DRC: Kongo Central** | 1689 | 0.2 | 0.1, 0.4 | 0.2 | 0.1, 0.4 | 0.2 | 0.1, 0.4 | 0.2 | 0.1, 0.4 |
| **Ethiopia** | 7361 | 0.1 | 0.1, 0.3 | 0.2 | 0.1, 0.4 | 0.3 | 0.1, 0.4 | 0.3 | 0.2, 0.5 |
| **Ghana** | 3683 | 1.3 | 0.8, 2.0 | 1.4 | 0.9, 2.1 | 1.5 | 1.0, 2.3 | 1.7 | 1.2, 2.5 |
| **India: Rajasthan** | 6034 | 0.1 | 0.0, 0.3 | 0.1 | 0.0, 0.3 | 0.1 | 0.0, 0.4 | 0.1 | 0.1, 0.4 |
| **Kenya** | 5876 | 0.7 | 0.4, 1.1 | 0.7 | 0.4, 1.2 | 1.5 | 1.1, 2.1 | **1.6** | **1.2, 2.2** |
| **Niger** | 3020 | 0.0 | 0.0, 0.1 | 0.0 | 0.0, 0.1 | 0.0 | 0.0, 0.1 | 0.0 | 0.0, 0.1 |
| **Nigeria: Anambra** | 1403 | 0.6 | 0.2, 1.9 | 0.8 | 0.3, 2.0 | 0.6 | 0.2, 1.9 | 0.8 | 0.3, 2.1 |
| **Nigeria:** **Kaduna** | 2855 | 0.1 | 0.0, 0.5 | 0.1 | 0.0, 0.5 | 0.2 | 0.1, 0.6 | 0.2 | 0.1, 0.6 |
| **Nigeria:** **Kano** | 1760 | 0 | -- | 0 | -- | 0 | -- | 0 | -- |
| **Nigeria:** **Lagos** | 1535 | 1.3 | 0.7, 2.4 | 1.4 | 0.8, 2.4 | 1.6 | 1.0, 2.7 | 1.7 | 1.0, 2.8 |
| **Nigeria:** **Nasarawa** | 1846 | 0.2 | 0.1, 0.6 | 0.2 | 0.1, 0.6 | 0.3 | 0.1, 0.9 | 0.3 | 0.1, 0.9 |
| **Nigeria:** **Rivers** | 1163 | 2.2 | 1.4, 3.6 | 2.2 | 1.4, 3.6 | 2.8 | 1.7, 4.6 | 2.8 | 1.7, 4.6 |
| **Nigeria: Taraba** | 818 | 0 | -- | 0 | -- | 0 | -- | 0 | -- |
| **Uganda** | 4225 | 0.4 | 0.2, 1.0 | 0.4 | 0.2, 1.0 | 0.6 | 0.3, 1.4 | 0.6 | 0.3, 1.4 |

Bolded values are statistically significant

T3 Emergency contraception use among all women based on Definition 1 and percentage point difference in the estimates using Definitions 2-4, by geography, bolded values are statistically significant

| **Country/ Geography** | **Emergency contraception use among all women, Definition 1**  **(%)** | **Percent point difference in estimates, compared to that based on Definition 1** | | |
| --- | --- | --- | --- | --- |
|  |  | **Definition 2** | **Definition 3** | **Definition 4** |
|  |  |  |  |  |
| **Burkina Faso** | 0.0 | 0.0 | 0.0 | 0.1 |
| **Côte d’Ivoire** | 1.2 | 0.1 | 0.4 | 0.4 |
| **DRC: Kinshasa** | 0.2 | 0.0 | 0.0 | 0.0 |
| **DRC: Kongo Central** | 1.4 | 0.1 | 0.3 | 0.4 |
| **Ethiopia** | 0.1 | 0.0 | 0.1 | 0.2 |
| **Ghana** | 1.3 | 0.2 | 0.3 | 0.4 |
| **India: Rajasthan** | 0.1 | 0.0 | 0.0 | 0.1 |
| **Kenya** | 0.7 | 0.0 | 0.9 | **0.9** |
| **Niger** | 0.0 | 0.0 | 0.0 | 0.0 |
| **Nigeria: Anambra** | 0.6 | 0.2 | 0.0 | 0.2 |
| **Nigeria: Kaduna** | 0.1 | 0.0 | 0.0 | 0.0 |
| **Nigeria: Kano** | 0.0 | 0.0 | 0.0 | 0.0 |
| **Nigeria: Lagos** | 1.3 | 0.1 | 0.3 | 0.4 |
| **Nigeria: Nasarawa** | 0.2 | 0.0 | 0.2 | 0.2 |
| **Nigeria: Rivers** | 2.2 | 0.0 | 0.6 | 0.6 |
| **Nigeria: Taraba** | 0.0 | 0.0 | 0.0 | 0.0 |
| **Uganda** | 0.4 | 0.0 | 0.2 | 0.2 |

Bolded values are statistically significant

T4 Emergency contraception use based on Definitions 1-4, by subgroup and geography (%), bolded values are statistically significant increases when compared to Definition 1

|  |  |  | **Definition 1** | | **Definition 2** | | **Definition 3** | | **Definition 4** | |
| --- | --- | --- | --- | --- | --- | --- | --- | --- | --- | --- |
| **Country/ Geography** | **Subgroup** | **N** | **Percent** | **Confidence Interval** | **Percent** | **Confidence Interval** | **Percent** | **Confidence Interval** | **Percent** | **Confidence Interval** |
| **Burkina Faso** | *Married* | 2413 | 0.0 | 0.0, 0.1 | 0.1 | 0.0, 0.3 | 0.0 | 0.0, 0.1 | 0.1 | 0.0, 0.3 |
|  | *Unmarried Sexually Active* | 429 | 0.3 | 0.0, 1.5 | 0.3 | 0.0, 1.5 | 0.5 | 0.1, 2.2 | 0.5 | 0.1, 2.2 |
|  | *Under 20* | 792 | 0.0 | 0.0, 0.3 | 0.0 | 0.0, 0.3 | 0.0 | 0.0, 0.3 | 0.0 | 0.0, 0.3 |
|  | *Under 25* | 1430 | 0.1 | 0.0, 0.3 | 0.1 | 0.0, 0.3 | 0.1 | 0.0, 0.3 | 0.1 | 0.0, 0.3 |
|  | *35 and Over* | 965 | 0 | –– | 0 | 0, 0.7 | 0 | –– | 0 | 0, 0.7 |
| **Côte d’Ivoire** | *Married* | 1775 | 0.7 | 0.3, 1.5 | 0.7 | 0.3, 1.5 | 0.8 | 0.4, 1.6 | 0.8 | 0.4, 1.6 |
|  | *Unmarried Sexually Active* | 545 | 4.8 | 2.6, 8.8 | 5.3 | 2.7, 10.3 | 5.8 | 3.5, 9.6 | 6.3 | 3.6, 11.0 |
|  | *Under 20* | 574 | 1.5 | 0.6, 3.5 | 1.5 | 0.6, 3.5 | 1.5 | 0.6, 3.5 | 1.5 | 0.6, 3.5 |
|  | *Under 25* | 1103 | 1.8 | 0.9, 3.5 | 1.8 | 0.9, 3.5 | 1.9 | 1.0, 3.7 | 1.9 | 1.0, 3.7 |
|  | *35 and Over* | 708 | 0.5 | 0.1, 1.8 | 0.5 | 0.1, 1.8 | 0.5 | 0.2, 1.8 | 0.5 | 0.2, 1.8 |
| **DRC: Kinshasa** | *Married* | 1166 | 0.4 | 0.2, 0.9 | 0.4 | 0.2, 0.9 | 0.6 | 0.3, 1.3 | 0.6 | 0.3, 1.3 |
|  | *Unmarried Sexually Active* | 680 | 3.8 | 2.3, 6.1 | 4.1 | 2.6, 6.5 | 4.8 | 3.0, 7.5 | 5.1 | 3.3, 7.8 |
|  | *Under 20* | 566 | 0.7 | 0.3, 1.9 | 0.7 | 0.3, 1.9 | 0.8 | 0.3, 2.1 | 0.8 | 0.3, 2.1 |
|  | *Under 25* | 1079 | 1.5 | 0.8, 2.9 | 1.5 | 0.8, 2.9 | 1.7 | 0.9, 3.2 | 1.7 | 0.9, 3.2 |
|  | *35 and Over* | 683 | 0.7 | 0.3, 1.8 | 0.7 | 0.3, 1.8 | 1.0 | 0.5, 2.1 | 1.0 | 0.5, 2.1 |
| **DRC: Kongo Central** | *Married* | 1094 | 0.1 | 0.0, 0.4 | 0.2 | 0.1, 0.4 | 0.1 | 0.0, 0.4 | 0.2 | 0.1, 0.4 |
|  | *Unmarried Sexually Active* | 337 | 0.4 | 0.1, 1.5 | 0.4 | 0.1, 1.5 | 0.4 | 0.1, 1.5 | 0.4 | 0.1, 1.5 |
|  | *Under 20* | 373 | 0.0 | 0.0, 0.1 | 0.0 | 0.0, 0.1 | 0.0 | 0.0, 0.1 | 0.0 | 0.0, 0.1 |
|  | *Under 25* | 635 | 0.1 | 0.0, 0.4 | 0.1 | 0.0, 0.4 | 0.1 | 0.0, 0.4 | 0.1 | 0.0, 0.4 |
|  | *35 and Over* | 527 | 0.1 | 0, 0.6 | 0.1 | 0, 0.6 | 0.1 | 0, 0.6 | 0.1 | 0, 0.6 |
| **Ethiopia** | *Married* | 4340 | 0.0 | 0.0, 0.1 | 0.0 | 0.0, 0.1 | 0.1 | 0.0, 0.2 | 0.1 | 0.1, 0.2 |
|  | *Unmarried Sexually Active* | 493 | 2.1 | 0.8, 5.2 | 2.5 | 1.0, 6.3 | 3.6 | 1.9, 6.7 | 4.1 | 2.1, 7.6 |
|  | *Under 20* | 1768 | 0.2 | 0.1, 0.5 | 0.2 | 0.1, 0.5 | 0.2 | 0.1, 0.6 | 0.2 | 0.1, 0.6 |
|  | *Under 25* | 3101 | 0.2 | 0.1, 0.4 | 0.2 | 0.1, 0.7 | 0.4 | 0.2, 0.7 | 0.4 | 0.2, 0.8 |
|  | *35 and Over* | 1810 | 0.0 | 0, 0.1 | 0.0 | 0, 0.1 | 0.0 | 0, 0.1 | 0.0 | 0, 0.1 |
| **Ghana** | *Married* | 2107 | 1.1 | 0.6, 2.0 | 1.3 | 0.7, 2.1 | 1.3 | 0.8, 2.2 | 1.5 | 0.9, 2.4 |
|  | *Unmarried Sexually Active* | 709 | 3.1 | 1.8, 5.2 | 3.5 | 2.1, 5.8 | 3.7 | 2.3, 5.9 | 4.2 | 2.7, 6.5 |
|  | *Under 20* | 708 | 1.1 | 0.5, 2.4 | 1.2 | 0.6, 2.5 | 1.1 | 0.5, 2.4 | 1.2 | 0.6, 2.5 |
|  | *Under 25* | 1386 | 1.5 | 0.9, 2.5 | 1.8 | 1.1, 2.9 | 1.7 | 1.0, 2.8 | 2.0 | 1.2, 3.2 |
|  | *35 and Over* | 1103 | 0.6 | 0.2, 1.8 | 0.6 | 0.2, 1.8 | 0.6 | 0.2, 1.8 | 0.6 | 0.2, 1.8 |
| **India: Rajasthan** | *Married* | 4554 | 0.1 | 0.0, 0.4 | 0.1 | 0.0, 0.4 | 0.2 | 0.1, 0.5 | 0.2 | 0.1, 0.5 |
|  | *Unmarried Sexually Active* | 52 | 0 | –– | 0 | –– | 0 | –– | 0 | –– |
|  | *Under 20* | 1173 | 0 | –– | 0 | –– | 0 | –– | 0 | –– |
|  | *Under 25* | 2337 | 0.2 | 0.0, 0.9 | 0.2 | 0.0, 0.9 | 0.2 | 0.0, 0.8 | 0.2 | 0.0, 0.8 |
|  | *35 and Over* | 1784 | 0.1 | 0, 0.4 | 0.1 | 0, 0.3 | 0.1 | 0, 0.4 | 0.1 | 0, 0.3 |
| **Kenya** | *Married* | 3404 | 0.1 | 0.1, 0.3 | 0.2 | 0.1, 0.3 | 0.4 | 0.2, 0.7 | 0.4 | 0.2, 0.7 |
|  | *Unmarried Sexually Active* | 1065 | 2.6 | 1.4, 4.6 | 2.7 | 1.5, 5.0 | 6.4 | 4.6, 8.7 | **6.5** | **4.7, 9.0** |
|  | *Under 20* | 1255 | 0.8 | 0.4, 1.7 | 0.8 | 0.4, 1.7 | 1.6 | 0.9, 2.7 | 1.6 | 0.9, 2.7 |
|  | *Under 25* | 2372 | 1.1 | 0.6, 2.0 | 1.1 | 0.6, 2.0 | 2.7 | 1.9, 3.8 | 2.7 | 1.9, 3.8 |
|  | *35 and Over* | 1556 | 0.1 | 0, 0.3 | 0.1 | 0, 0.3 | 0.1 | 0, 0.3 | 0.1 | 0, 0.3 |
| **Niger** | *Married* | 2161 | 0.0 | 0.0, 0.1 | 0.0 | 0.0, 0.1 | 0.0 | 0.0, 0.1 | 0.0 | 0.0, 0.1 |
|  | *Unmarried Sexually Active* | 99 | 0 | –– | 0 | –– | 0 | –– | 0 | –– |
|  | *Under 20* | 679 | 0 | –– | 0 | –– | 0 | –– | 0 | –– |
|  | *Under 25* | 1232 | 0.0 | 0.0, 0.2 | 0.0 | 0.0, 0.2 | 0.0 | 0.0, 0.2 | 0.0 | 0.0, 0.2 |
|  | *35 and Over* | 852 | 0.0 | 0, 0.1 | 0.0 | 0, 0.1 | 0.0 | 0, 0.1 | 0.0 | 0, 0.1 |
| **Nigeria: Anambra** | *Married* | 703 | 1.0 | 0.3, 3.7 | 1.0 | 0.3, 3.7 | 1.1 | 0.3, 3.7 | 1.1 | 0.3, 3.7 |
|  | *Unmarried Sexually Active* | 230 | 0.5 | 0.1, 3.1 | 1.5 | 0.3, 6.0 | 0.5 | 0.1, 3.1 | 1.5 | 0.3, 6.0 |
|  | *Under 20* | 303 | 0 | –– | 0 | –– | 0 | –– | 0 | –– |
|  | *Under 25* | 524 | 0.3 | 0.0, 1.6 | 0.3 | 0.0, 1.6 | 0.3 | 0.0, 1.6 | 0.3 | 0.0, 1.6 |
|  | *35 and Over* | 436 | 0.8 | 0.2, 3.1 | 0.8 | 0.2, 3.1 | 0.8 | 0.2, 3.1 | 0.8 | 0.2, 3.1 |
| **Nigeria: Kaduna** | *Married* | 2217 | 0.1 | 0.0, 0.3 | 0.1 | 0.0, 0.3 | 0.1 | 0.0, 0.3 | 0.1 | 0.0, 0.3 |
|  | *Unmarried Sexually Active* | 132 | 2.3 | 0.6, 8.1 | 2.3 | 0.6, 8.1 | 2.3 | 0.6, 8.1 | 2.3 | 0.6, 8.1 |
|  | *Under 20* | 700 | 0 | –– | 0 | –– | **0.1** | **0.0, 0.9** | **0.1** | **0.0, 0.9** |
|  | *Under 25* | 1251 | 0.1 | 0.0, 0.6 | 0.1 | 0.0, 0.6 | 0.2 | 0.0, 0.7 | 0.2 | 0.0, 0.7 |
|  | *35 and Over* | 664 | 0 | –– | 0 | –– | 0 | –– | 0 | –– |
| **Nigeria: Kano** | *Married* | 1280 | 0 | –– | 0 | –– | 0 | –– | 0 | –– |
|  | *Unmarried Sexually Active* | 40 | 0 | –– | 0 | –– | 0 | –– | 0 | –– |
|  | *Under 20* | 419 | 0 | –– | 0 | –– | 0 | –– | 0 | –– |
|  | *Under 25* | 744 | 0 | –– | 0 | –– | 0 | –– | 0 | –– |
|  | *35 and Over* | 471 | 0 | –– | 0 | –– | 0 | –– | 0 | –– |
| **Nigeria: Lagos** | *Married* | 1001 | 0.8 | 0.4, 1.9 | 0.8 | 0.4, 1.9 | 0.9 | 0.4, 2.0 | 0.9 | 0.4, 2.0 |
|  | *Unmarried Sexually Active* | 181 | 4.9 | 2.2, 10.4 | 5.7 | 2.8, 11.1 | 5.9 | 3.0, 11.4 | 6.7 | 3.6, 12.1 |
|  | *Under 20* | 218 | 0.8 | 0.2, 3.2 | 1.4 | 0.5, 4.1 | 1.8 | 0.6, 5.3 | 2.3 | 0.9, 5.8 |
|  | *Under 25* | 421 | 2.6 | 1.3, 5.3 | 2.9 | 1.5, 5.5 | 3.1 | 1.6, 5.8 | 3.4 | 1.9, 6.0 |
|  | *35 and Over* | 551 | 0.7 | 0.3, 1.8 | 0.7 | 0.3, 1.8 | 0.8 | 0.3, 2 | 0.8 | 0.3, 2 |
| **Nigeria: Nasarawa** | *Married* | 1187 | 0.0 | 0.0, 0.1 | 0.0 | 0.0, 0.1 | 0.0 | 0.0, 0.1 | 0.0 | 0.0, 0.1 |
|  | *Unmarried Sexually Active* | 244 | 1.3 | 0.4, 4.3 | 1.3 | 0.4, 4.3 | 2.5 | 1.0, 6.1 | 2.5 | 1.0, 6.1 |
|  | *Under 20* | 422 | 0.2 | 0.0, 0.9 | 0.2 | 0.0, 0.9 | 0.7 | 0.2, 2.7 | 0.7 | 0.2, 2.7 |
|  | *Under 25* | 763 | 0.2 | 0.1, 0.8 | 0.2 | 0.1, 0.8 | 0.5 | 0.2, 1.5 | 0.5 | 0.2, 1.5 |
|  | *35 and Over* | 439 | 0 | –– | 0 | –– | 0 | –– | 0 | –– |
| **Nigeria: Rivers** | *Married* | 670 | 1.5 | 0.7, 3.0 | 1.5 | 0.7, 3.0 | 1.9 | 1.0, 3.7 | 1.9 | 1.0, 3.7 |
|  | *Unmarried Sexually Active* | 249 | 6.5 | 3.7, 11.3 | 6.5 | 3.7, 11.3 | 8.1 | 4.7, 13.7 | 8.1 | 4.7, 13.7 |
|  | *Under 20* | 194 | 1.9 | 0.5, 6.6 | 1.9 | 0.5, 6.6 | 1.9 | 0.5, 6.6 | 1.9 | 0.5, 6.6 |
|  | *Under 25* | 403 | 2.7 | 1.4, 5.1 | 2.7 | 1.4, 5.1 | 3.3 | 1.6, 6.5 | 3.3 | 1.6, 6.5 |
|  | *35 and Over* | 359 | 0.6 | 0.2, 1.9 | 0.6 | 0.2, 1.9 | 0.8 | 0.2, 2.5 | 0.8 | 0.2, 2.5 |
| **Nigeria: Taraba** | *Married* | 569 | 0 | –– | 0 | –– | 0 | –– | 0 | –– |
|  | *Unmarried Sexually Active* | 113 | 0 | –– | 0 | –– | 0 | –– | 0 | –– |
|  | *Under 20* | 171 | 0 | –– | 0 | –– | 0 | –– | 0 | –– |
|  | *Under 25* | 320 | 0 | –– | 0 | –– | 0 | –– | 0 | –– |
|  | *35 and Over* | 207 | 0 | –– | 0 | –– | 0 | –– | 0 | –– |
| **Uganda** | *Married* | 2674 | 0.1 | 0.0, 0.3 | 0.1 | 0.0, 0.3 | 0.1 | 0.0, 0.3 | 0.1 | 0.0, 0.3 |
|  | *Unmarried Sexually Active* | 655 | 2.4 | 1.1, 5.2 | 2.4 | 1.1, 5.2 | 3.4 | 1.6, 7.3 | 3.4 | 1.6, 7.3 |
|  | *Under 20* | 919 | 0.4 | 0.1, 2.0 | 0.4 | 0.1, 2.0 | 0.5 | 0.1, 2.0 | 0.5 | 0.1, 2.0 |
|  | *Under 25* | 1813 | 0.7 | 0.3, 1.5 | 0.7 | 0.3, 1.5 | 1.0 | 0.4, 2.3 | 1.0 | 0.4, 2.3 |
|  | *35 and Over* | 1087 | 0.1 | 0, 0.5 | 0.1 | 0, 0.5 | 0.1 | 0, 0.5 | 0.1 | 0, 0.5 |

Bolded values are statistically significant

T5 Emergency contraception use based on Definition 1 and percentage point difference in the estimates using Definitions 2-4, by subgroup and geography

| **Country/ Geography** | **Emergency contraception use among all women, Definition 1**  **(%)** | **Percent point difference in estimates, compared to that based on Definition 1** | | |
| --- | --- | --- | --- | --- |
| Subgroup |  | **Definition 2** | **Definition 3** | **Definition 4** |
| **Burkina Faso** | | | | |
| Married | 0.0 | 0.0 | 0.0 | 0.1 |
| Unmarried Sexually Active | 0.3 | 0.0 | 0.3 | 0.3 |
| Under 20 | 0.0 | 0.0 | 0.0 | 0.0 |
| Under25 | 0.1 | 0.0 | 0.0 | 0.0 |
| 35 and Over | 0.0 | 0.1 | 0.0 | 0.1 |
| **Côte d’Ivoire** | | | | |
| Married | 0.7 | 0.0 | 0.1 | 0.1 |
| Unmarried Sexually Active | 4.8 | 0.5 | 1.0 | 1.5 |
| Under 20 | 1.5 | 0.0 | 0.0 | 0.0 |
| Under25 | 1.8 | 0.0 | 0.1 | 0.1 |
| 35 and Over | 0.5 | 0.0 | 0.1 | 0.1 |
| **DRC – Kinshasa** | | | | |
| Married | 0.4 | 0.0 | 0.2 | 0.2 |
| Unmarried Sexually Active | 3.8 | 0.3 | 1.0 | 1.3 |
| Under 20 | 0.7 | 0.0 | 0.1 | 0.1 |
| Under25 | 1.5 | 0.0 | 0.2 | 0.2 |
| 35 and Over | 0.7 | 0.0 | 0.3 | 0.3 |
| **DRC – Kongo Central** | | | | |
| Married | 0.1 | 0.0 | 0.0 | 0.0 |
| Unmarried Sexually Active | 0.4 | 0.0 | 0.0 | 0.0 |
| Under 20 | 0.0 | 0.0 | 0.0 | 0.0 |
| Under25 | 0.1 | 0.0 | 0.0 | 0.0 |
| 35 and Over | 0.1 | 0.0 | 0.0 | 0.0 |
| **Ethiopia** | | | | |
| Married | 0.0 | 0.0 | 0.1 | 0.1 |
| Unmarried Sexually Active | 2.1 | 0.4 | 1.5 | 2.0 |
| Under 20 | 0.2 | 0.0 | 0.0 | 0.0 |
| Under25 | 0.2 | 0.1 | 0.2 | 0.3 |
| 35 and Over | 0.0 | 0.0 | 0.0 | 0.0 |
| **Ghana** | | | | |
| Married | 1.1 | 0.1 | 0.2 | 0.4 |
| Unmarried Sexually Active | 3.1 | 0.5 | 0.7 | 1.1 |
| Under 20 | 1.1 | 0.1 | 0.0 | 0.1 |
| Under25 | 1.5 | 0.3 | 0.2 | 0.5 |
| 35 and Over | 0.6 | 0.1 | 0.0 | 0.1 |
| **Rajasthan** | | | | |
| Married | 0.1 | 0.0 | 0.1 | 0.1 |
| Unmarried Sexually Active | 0.0 | 0.0 | 0.0 | 0.0 |
| Under 20 | 0.0 | 0.0 | 0.0 | 0.0 |
| Under25 | 0.2 | 0.0 | 0.0 | 0.0 |
| 35 and Over | 0.1 | 0.0 | 0.0 | 0.0 |
| **Kenya** | | | | |
| Married | 0.1 | 0.0 | 0.3 | 0.3 |
| Unmarried Sexually Active | 2.6 | 0.2 | 3.8 | **4.0** |
| Under 20 | 0.8 | 0.0 | 0.7 | 0.7 |
| Under25 | 1.1 | 0.0 | 1.6 | 1.6 |
| 35 and Over | 0.1 | 0.0 | 0.0 | 0.0 |
| **Niger** | | | | |
| Married | 0.0 | 0.0 | 0.0 | 0.0 |
| Unmarried Sexually Active | 0.0 | 0.0 | 0.0 | 0.0 |
| Under 20 | 0.0 | 0.0 | 0.0 | 0.0 |
| Under25 | 0.0 | 0.0 | 0.0 | 0.0 |
| 35 and Over | 0.0 | 0.0 | 0.0 | 0.0 |
| **Nigeria: Anambra** | | | | |
| Married | 1.0 | 0.0 | 0.1 | 0.1 |
| Unmarried Sexually Active | 0.5 | 0.9 | 0.0 | 0.9 |
| Under 20 | 0.0 | 0.0 | 0.0 | 0.0 |
| Under25 | 0.3 | 0.0 | 0.0 | 0.0 |
| 35 and Over | 0.8 | 0.0 | 0.0 | 0.0 |
| **Nigeria: Kaduna** | | | | |
| Married | 0.1 | 0.0 | 0.0 | 0.0 |
| Unmarried Sexually Active | 2.3 | 0.0 | 0.0 | 0.0 |
| Under 20 | 0.0 | 0.0 | **0.1** | **0.1** |
| Under25 | 0.1 | 0.0 | 0.1 | 0.1 |
| 35 and Over | 0.0 | 0.0 | 0.0 | 0.0 |
| **Nigeria: Kano** | | | | |
| Married | 0.0 | 0.0 | 0.0 | 0.0 |
| Unmarried Sexually Active | 0.0 | 0.0 | 0.0 | 0.0 |
| Under 20 | 0.0 | 0.0 | 0.0 | 0.0 |
| Under25 | 0.0 | 0.0 | 0.0 | 0.0 |
| 35 and Over | 0.0 | 0.0 | 0.0 | 0.0 |
| **Nigeria: Lagos** | | | | |
| Married | 0.8 | 0.0 | 0.1 | 0.1 |
| Unmarried Sexually Active | 4.9 | 0.7 | 1.0 | 1.8 |
| Under 20 | 0.8 | 0.6 | 0.9 | 1.5 |
| Under25 | 2.6 | 0.3 | 0.5 | 0.8 |
| 35 and Over | 0.7 | 0.0 | 0.2 | 0.2 |
| **Nigeria: Nasarawa** | | | | |
| Married | 0.0 | 0.0 | 0.0 | 0.0 |
| Unmarried Sexually Active | 1.3 | 0.0 | 1.1 | 1.1 |
| Under 20 | 0.2 | 0.0 | 0.5 | 0.5 |
| Under25 | 0.2 | 0.0 | 0.3 | 0.3 |
| 35 and Over | 0.0 | 0.0 | 0.0 | 0.0 |
| **Nigeria: Rivers** | | | | |
| Married | 1.5 | 0.0 | 0.4 | 0.4 |
| Unmarried Sexually Active | 6.5 | 0.0 | 1.6 | 1.6 |
| Under 20 | 1.9 | 0.0 | 0.0 | 0.0 |
| Under25 | 2.7 | 0.0 | 0.6 | 0.6 |
| 35 and Over | 0.6 | 0.0 | 0.2 | 0.2 |
| **Nigeria: Taraba** | | | | |
| Married | 0.0 | 0.0 | 0.0 | 0.0 |
| Unmarried Sexually Active | 0.0 | 0.0 | 0.0 | 0.0 |
| Under 20 | 0.0 | 0.0 | 0.0 | 0.0 |
| Under25 | 0.0 | 0.0 | 0.0 | 0.0 |
| 35 and Over | 0.0 | 0.0 | 0.0 | 0.0 |
| **Uganda** | | | | |
| Married | 0.1 | 0.0 | 0.0 | 0.0 |
| Unmarried Sexually Active | 2.4 | 0.0 | 1.0 | 1.0 |
| Under 20 | 0.4 | 0.0 | 0.0 | 0.0 |
| Under25 | 0.7 | 0.0 | 0.3 | 0.3 |
| 35 and Over | 0.1 | 0.0 | 0.0 | 0.0 |

Bolded values are statistically significant
